# Arthritis progressors have a decreased frequency of circulating autoreactive T cells during the at-risk phase of rheumatoid arthritis

**DOI:** 10.1136/rmdopen-2024-004510

**Published:** 2024-11-18

**Authors:** Sara Turcinov, Ravi Kumar Sharma, Charlotte De Vries, Alexandra Cîrciumaru, Christina Gerstner, Linda Mathsson-Alm, Bruno Raposo, Anatoly Dubnovitsky, Lars Rönnblom, William W Kwok, Karine Chemin, Vivianne Malmström, Aase Hensvold

**Affiliations:** 1 Division of Rheumatology, Department of Medicine Solna, Center for Molecular Medicine, Karolinska Institutet, Solna, Sweden; 2 Theme of Inflammation and Ageing, Medical Unit Gastro, Derma, Rheuma, Karolinska University Hospital, Solna, Sweden; 3 Center for Rheumatology, Academic Specialist Center, Stockholm Health Services, Region Stockholm, Stockholm, Sweden; 4 Thermo Fischer Scientific, Uppsala, Sweden; 5 Department of Medical Sciences, Rheumatology, Uppsala University, Uppsala, Sweden; 6 Benaroya Research Institute at Virginia Mason, Seattle, Washington, USA

**Keywords:** T Cells, Arthritis, Anti-Citrullinated Protein Antibodies

## Abstract

**Objectives:**

The aim of this study was to combine deep T cell phenotyping with assessment of citrulline-reactive CD4+T cells in the pre-rheumatoid arthritis (RA) phase.

**Methods:**

20 anti-CCP2 positive individuals (HLA-DRB1*04:01) presenting musculoskeletal complaints without clinical or ultrasound signs of synovitis; 10 arthritis progressors and 10 matched non-arthritis progressors were included. Longitudinal samples (1–3 time points) of peripheral blood mononuclear cells were assessed using HLA-class II tetramers with 12 different citrullinated candidate autoantigens combined in a >20-colour spectral flow cytometry panel.

**Results:**

The baseline CD4+T cell phenotype was similar between individuals who progressed to arthritis (ie, in the pre-RA phase) and the non-progressors, when studying markers associated with Th1, Th17, T-peripheral and T-regulatory cells as well as with T-cell activation. Citrulline-reactive CD4+T cells were present in both groups but at significantly lower frequency in the progressor group. CD4+T cells specific for citrullinated tenascin-C were the most frequently observed among the progressors, and their frequencies diminished during follow-up that is, closer to arthritis onset. Notably, PD-1 and CD95 expression on the memory cit-tenascin-C-specific T cells in this group indicated repeated antigen exposure.

**Conclusions:**

Our data lend support to citrullinated tenascin-C as an interesting T cell antigen in RA. Moreover, lower frequency of circulating citrulline-specific cells in arthritis progressing individuals suggest an initiated homing of these cells to the joints and/or their associated lymph nodes in the pre-RA phase and a possible window of opportunity for therapeutic preventive interventions.

**WHAT IS ALREADY KNOWN ON THIS TOPIC**

- Autoreactive CD4+T cells recognising citrullinated (cit) peptides are rare but present in the circulation and synovial fluid of patients with rheumatoid arthritis, whereas there are yet limited data for the at-risk phase leading up to disease onset.

**WHAT THIS STUDY ADDS**

- This study reveals that individuals who develop arthritis differs from non-yet-progressors by having a lower frequency of circulating autoreactive CD4+T cells in the at-risk phase of the disease. Cit-tenascin-C-specific T cells are the most frequent at this stage, priorly only shown in established rheumatoid arthritis.

**HOW THIS STUDY MIGHT AFFECT RESEARCH, PRACTICE OR POLICY**

- Unravelling the occurrence and phenotype of autoreactive CD4+T cells prior to disease onset present a window of opportunity for future preventive interventions.

## Introduction

 The development of rheumatoid arthritis (RA) is a process where loss of self-tolerance is thought to occur over time in genetically susceptible individuals following different environmental triggers.^1 2^ Antibodies towards citrullinated proteins (ACPA) are found in the majority of RA patients and have been shown to appear many years before disease onset.^3 4^ This phase of systemic autoimmunity (occurrence of circulating autoantibodies) prior to diagnosis of RA can in turn be followed by musculoskeletal complaints without clinical manifestation of arthritis, representing a time window where individuals are at risk of developing future disease.^5^ Notably, whereas about 40% of ACPA-positive risk individuals presenting musculoskeletal symptoms develop arthritis during a 3-year follow-up period, a large proportion of at-risk individuals do not progress or progress at a much later time point.^6^,^8^

The strongest genetic risk loci for ACPA-positive RA are located at chromosome 6 in the HLA-class II locus,^9 10^ where HLA-DRB1*04:01 is one of the alleles conveying this risk.^11^ Antigen-specific CD4+T cells, recognising citrullinated self-peptides (eg, citrullinated α-enolase, vimentin and tenascin-C) presented by HLA-DRB1*04:01, constitute a small population of T cells in both peripheral blood^12^,^17^ and synovial fluid of RA patients^17 18^ at time of diagnosis and during the disease course. Whereas virus-specific CD4+T cells in peripheral blood are primarily memory cells, citrulline-specific T cells can be seen both in the memory and the naïve compartment in patients with RA.^13 16 18^

There is a great interest to understand the immunological events that lead to the development of RA, and possibilities to prevent disease by T cell targeting therapy have been investigated in treatment trials (APPIPRA, ARIAA).^19 20^ Nevertheless, there are yet limited cellular studies on the risk-RA phase of the disease, particularly those assessing the occurrence of autoreactive (citrulline-specific) T cells, or in-depth phenotype of the overall T cell compartment during this stage. However, a recent study showed an increase of citrulline-specific T cells in at-risk individuals in comparison to controls^21^ and assessment of major T cell subsets has shown a decreased frequency of, for example, naïve CD4+T cells and Tregs in individuals who progress to RA.^22 23^

Making use of the increased deep phenotyping potential of full spectrum flow cytometry, we here combined this technique with a previously demonstrated approach to phenotype and monitor citrulline-specific CD4+T cells based on multi peptide-HLA class II tetramer staining and surface markers expression.^16^

The aim of this study was to investigate the presence of autoreactive CD4+T cells recognising citrullinated epitopes and the overall T cell phenotype in longitudinal peripheral blood samples captured prospectively before disease onset in individuals at high risk of developing RA. Thus, increasing the understanding of the immunological events leading up to disease onset and identifying possible targets for therapeutic preventive interventions.

## Material and methods

### T cell study within the Karolinska Risk-RA cohort

The study individuals were included from the Karolinska Risk-RA prospective cohort (n=254) by having (1) at least one HLA-DRB1*04:01 allele and (2) >30 million cryopreserved peripheral blood mononuclear cells (PBMCs) at baseline and follow-up time points. 10 individuals with known arthritis progression were eligible and randomly matched by ten non-progressors based on number of samples, sex and age. Individuals who was included have been referred to the rheumatology clinic with ACPA-positivity (by anti-CCP2 IgG test; anti-cyclic citrullinated peptide test) and musculoskeletal complaints but were lacking clinical and ultrasound detected synovitis on rheumatological assessment. Clinical data were collected by November 2022 when the individuals had been followed for at least 36 months, or until progression to clinically manifest arthritis. Blood samples were taken at scheduled yearly visits and on-demand visits.^8^ Median time from inclusion to disease progression (clinical observation time) was 13 months (range 1–32) in the progressors, whereas the non-progressors had not yet developed arthritis during median 53 months (range 35–70) of follow-up (online supplemental figure 1A).

All individuals had PBMCs available from baseline sampling. Nine individuals in each group also had follow-up samples (one time point n=7, two time points n=2). In the progressor group, seven of these samples were available from arthritis onset (end-point). Duration between baseline sampling and end-point or last follow-up sampling was in median 10 months (IQR 1–14) in the progressor group and 16 months (IQR 13–26) in the non-progressor group (online supplemental figure 1B).

**Figure 1:**
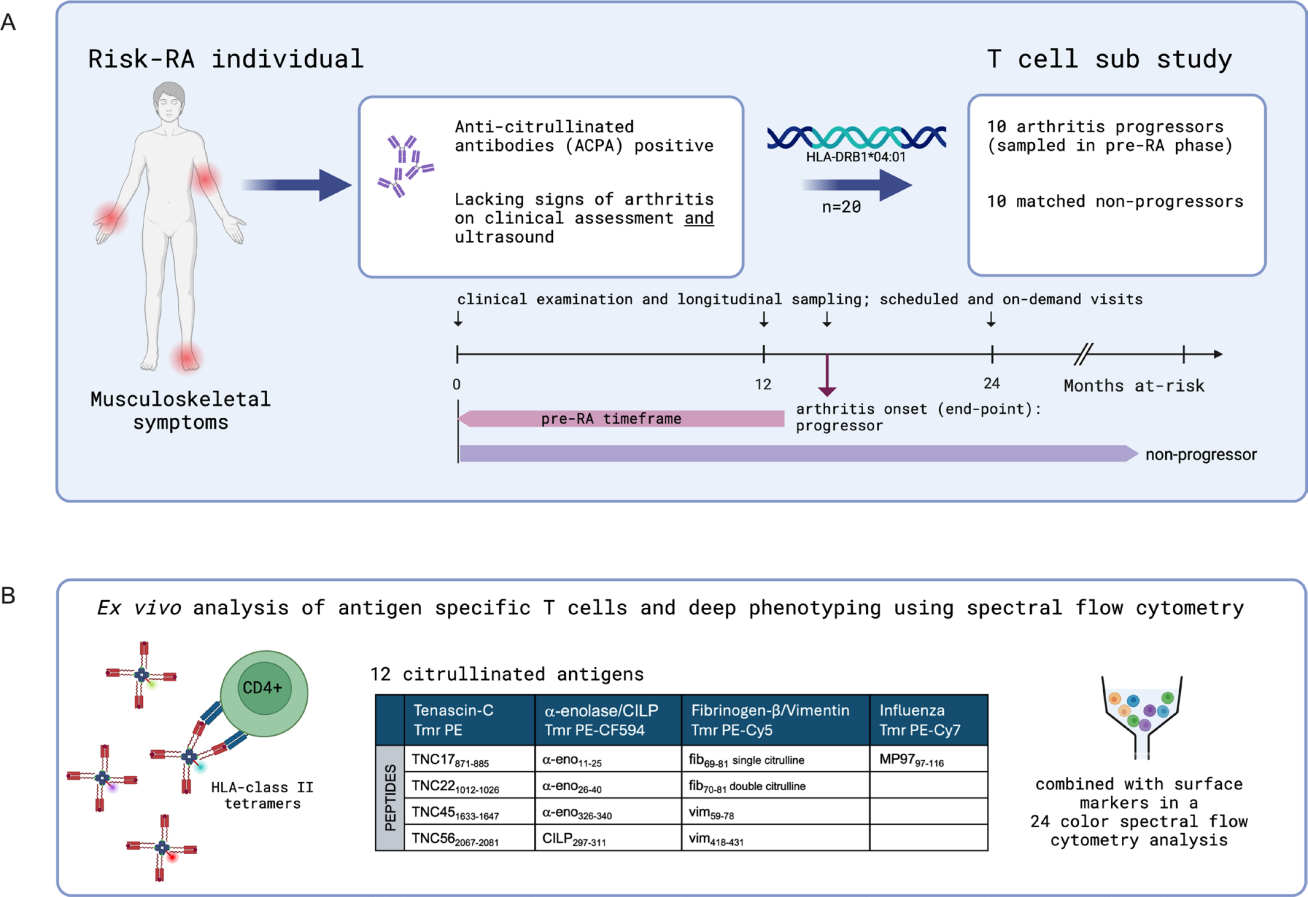
Study cohort and the use of ex vivo tetramers and deep phenotyping. (**A**) Individuals with musculoskeletal complaints and a positive anti-CCP2 test in primary healthcare but lacking signs of synovitis by rheumatological assessment were selected from the Karolinska Risk-RA cohort by having an HLA-DRB1*04:01 allele, >30 million peripheral blood mononuclear cells at 1–3 sample time points. Ten known arthritis progressors (sampled in the pre-RA phase) were randomly matched with 10 non-progressors. Individuals are followed to arthritis onset (end-point) or at least 36 months. (**B**) HLA-class II tetramers were assembled with citrullinated antigens and combined into tetramer pools; for example, four separate cit-TNC tetramers labelled with PE. Tetramer staining was combined into a 24-colour full spectrum flow cytometry panel to assess antigen specificity and phenotype of T cells. The figures were created with BioRender.com. RA, rheumatoid arthritis.

All arthritis cases except one fulfilled the American College of Rheumatology/European Alliance of Associations for Rheumatology 2010 classification criteria for RA. The overall study outline is depicted in figure 1A.

### Healthy donors

PBMCs from four HLA-DRB1*04:01 positive healthy donors (Uppsala Bioresource) were included as a control group for ex vivo assessment of antigen-specific CD4+T cells.

### Sample preparation and cryopreservation

PBMCs were separated using the Ficoll-Paque gradient method (GE Healthcare), frozen at −80°C in fetal bovine serum +10% DMSO (Merck-Sigma) overnight, followed by long-term storage in liquid nitrogen until sample assessment.

### Assembly of HLA-class II tetramers

The HLA-DRB1*04:01 monomers were produced and in vitro biotinylated as previously described,^24^ followed by endotoxin removal using Triton-X114.^25^ The biotinylated monomers were incubated with the individual peptides (citrullinated peptides, n=12; positive control: influenza-derived peptide, n=1, (online supplemental table 1), n-octyl-β-D-glucopyranoside and Pefabloc SC (Merck-Sigma) at 37°C for 72 hours. The peptide-loaded monomers were consecutively tetramerised using streptavidin-fluorochrome conjugates (SA-PE, SA-PE-Cy5, SA-PE-CF594 and SA-PE-Cy7 all BD Biosciences) forming 13 tetramers divided into 4 tetramer pools (figure 1B, online supplemental table 1).^24^

The specificity of peptide-HLA complexes was confirmed using the murine 58 TCRα-β- T cell line (58^−/−^),^26^ transfected with patient-derived TCRs recognising citrullinated fibrinogen β (fib_69−81_), citrullinated tenascin-C (TNC_1012−1026_) or influenza (MP97_97−116_) peptides, respectively, generated as per published protocol.^27^ These T cell lines have been engineered to express an NFAT-GFP reporter, which results in increased GFP expression on TCR stimulation, measured by flow cytometry. Briefly, anti-mouse CD3 or peptide loaded monomers were used for coating 48 well plates overnight at 4°C (100 µL coating volume, in PBS containing Ca^2+^). After discarding the supernatant, the 58^−/−^ cells were added to the coated plates and incubated for 48 hours, using anti-mouse CD3/CD28 (Biolegend) antibody stimulation as a positive control. The cells were then stained with viability dye (Live/Dead near IR) and assessed for GFP expression in the presence and absence of peptide-HLA complex stimulation, using flow cytometry^28^ (online supplemental figure 2).

### Ex vivo tetramer assessment

Cryopreserved PBMCs were thawed at 37°C in 1640-RPMI supplemented with 10% fetal bovine serum, penicillin/streptomycin (100U/100 ug/mL), 10 mM HEPES (all Merck-Sigma) and 2 mM L-glutamine (Gibco, ThermoFisher). Cells were incubated with protein kinase inhibitor dasatinib for 10 min at 37°C 5% CO_2_, followed by tetramer staining for 90 min at room temperature. Anti-PE microbeads (Miltenyi) were used for magnetic enrichment of tetramer labelled cells. Prior to this, 1% of the pre-enriched sample was set aside for phenotypic assessment and calculation of CD4+T cell frequencies per sample, allowing determination of a number of tetramer-positive cells per million CD4+T cells in the sample. Pre-enriched and post-enriched samples were stained with antibodies against surface receptors for 15 min at 20°C, followed by viability dye staining (20-colour panel; online supplemental table 2), for multiparameter phenotyping. Cells were acquired on a 4-laser CYTEK Aurora (Cytek Biosciences) with unmixing performed using SpectroFlo software (V.3.0.3). All samples were assessed during a limited time frame using the same batch of tetramers and antibodies. For each experiment, a minimum of two individuals were assessed in parallel, and all samples from an individual were run in the same experiment.

FlowJo (V.10.8.1, BD Biosciences) was used for analysis of flow cytometry data. A Boolean gating approach was used to exclude any cells recognising >1 antigen specificity, to avoid unspecific binding. The pre-enriched samples were used to determine background staining and set the tetramer gate for the tetramer-enriched sample. Cells defined as tetramer positive in the pre-enriched sample were also included for the subsequent phenotypic analysis.

### Surface marker antibodies and gating strategies

Dump negative (CD14+, CD16+, CD19+ cells and/or dead cells) but CD3+CD4+CD8− T cells (gating strategy in online supplemental figure 3) were included for downstream phenotyping and tetramer assessment (denoted CD4+ in results). Marker combinations used as a proxy to identify CD4+T cell subsets: Th1 (CXCR3+CCR6−), Th17 (CXCR3-CCR6+), Th1* (CXCR3+CCR6+),^29^ T-peripheral helper cells (Tph; CXCR5-PD-1hi), T-follicular helper cells (Tfh; CXCR5+), Treg (CD127-CD25+), Treg subsets (activated aTregs: CD127-CD25+CD45RA-CD25hi, naïve/resting rTregs: CD127-CD25+CD45RA+CD25 med^30^). Naïve CD4+T cells (CD28+CD45RA+), memory CD4+T cells (CD28+CD45RA− or CD28null) and CD28+CD45RAdim cells were assessed separately. Memory subsets included central memory (CM; CD45RA-CCR7+), effector memory (EM; CD45RA-CCR7−), CD45RA+EM (TEMRA; CD28nullCD45RA+). The gates from CD28nullCCR7± were used to assist CCR7 gating of CD28+CD45RA− CD4+T cells (online supplemental figure 4). The gating strategy for activation markers, CD25+, CD95+, CD137+, PD-1+, HLA-DR+, CD38 vs CD69 within the CD4+CD8− compartment, is found in online supplemental figure 5. Gates from the main CD4+CD8− population were applied to naïve and memory subsets, respectively, and in the same way, all gates were applied to the tetramer-positive cells for phenotyping.

### Microarray analysis of ACPA reactivities

Serum samples from baseline visit corresponding to the time point for the research PBMC samples were analysed for ACPA reactivities using a custom-made multiplex solid phase microarray platform (Thermo Fisher Scientific, ImmunoDiagnostics Division, Uppsala, Sweden), as described previously.^31^

### Statistics

Statistical analysis was made in GraphPad Prism (V.9.4.0). Median values are presented with range min-max or 1st–3rd IQR. Mann-Whitney U two-tailed test was used for unpaired, whereas Wilcoxon matched-pairs signed rank test (two tailed) was used for paired, non-parametrical data. Friedman test was used for multiple comparisons of paired non-parametric data and Kruskal-Wallis test for multiple comparisons of unpaired non-parametric data; both with Dunn’s multiple comparisons test to correct for multiple analyses. Usage of statistical method as indicated in figure legends. A p<0.05 was considered significant.

## Results

### Clinical characteristics

Clinical characteristics at baseline for the 20 individuals at high risk of developing RA are summarised in table 1. As an expected finding corresponding to the overall cohort,^8^ several baseline characteristics were different in those already known to progress to arthritis compared with the non-yet-progressors, such as anti-CCP2 levels, co-occurrence of rheumatoid factor (RF) and double shared epitope alleles, where seven progressors (70%) and four (40%) non-progressors had double shared epitope alleles. In the progressor group, there was a broader antibody reactivity to different citrullinated peptides (median 6, range 1–7) compared with the non-progressor group (median 1.5, range 0–9; online supplemental figure 6).

**Table 1:** Characteristics at inclusion of the Karolinska Risk-RA T cell cohort (n=20)

	Progressors, n=10	Non-progressors, n=10
Age, years, median (IQR)	50 (36–60)	45 (37–60)
Female, n (%)	4 (40)	8 (80)
Current smoker, n (%)	2 (20)	1 (10)
Anti-CCP2 levels*, median (IQR)	100 (17–100)	2.2 (1.0–60)
RF positive, n (%)	6 (60)	2 (20)
Double HLA-SE allele positive, n (%)	7 (70)	4 (40)

* Anti-CCP2 IgG levels. Clinical test results are normalizednormalised to the cut-off used in the assays, and the levels is are presented as number times increased to cut offcut-off at 1. 100 is the upper limit of detection for the anti-CCP2 assays used. Abbreviations: n, number; , interquartile range 1st-3rd; Anti-CCP, anti-cyclic citrullinated peptide antibodies; RF, rheumatoid factor.

Anti-CCPanti-cyclic citrullinated peptide antibodiesnnumberRFrheumatoid factor

### Cell subsets in the global CD4+ compartment at baseline

**Figure 2:**
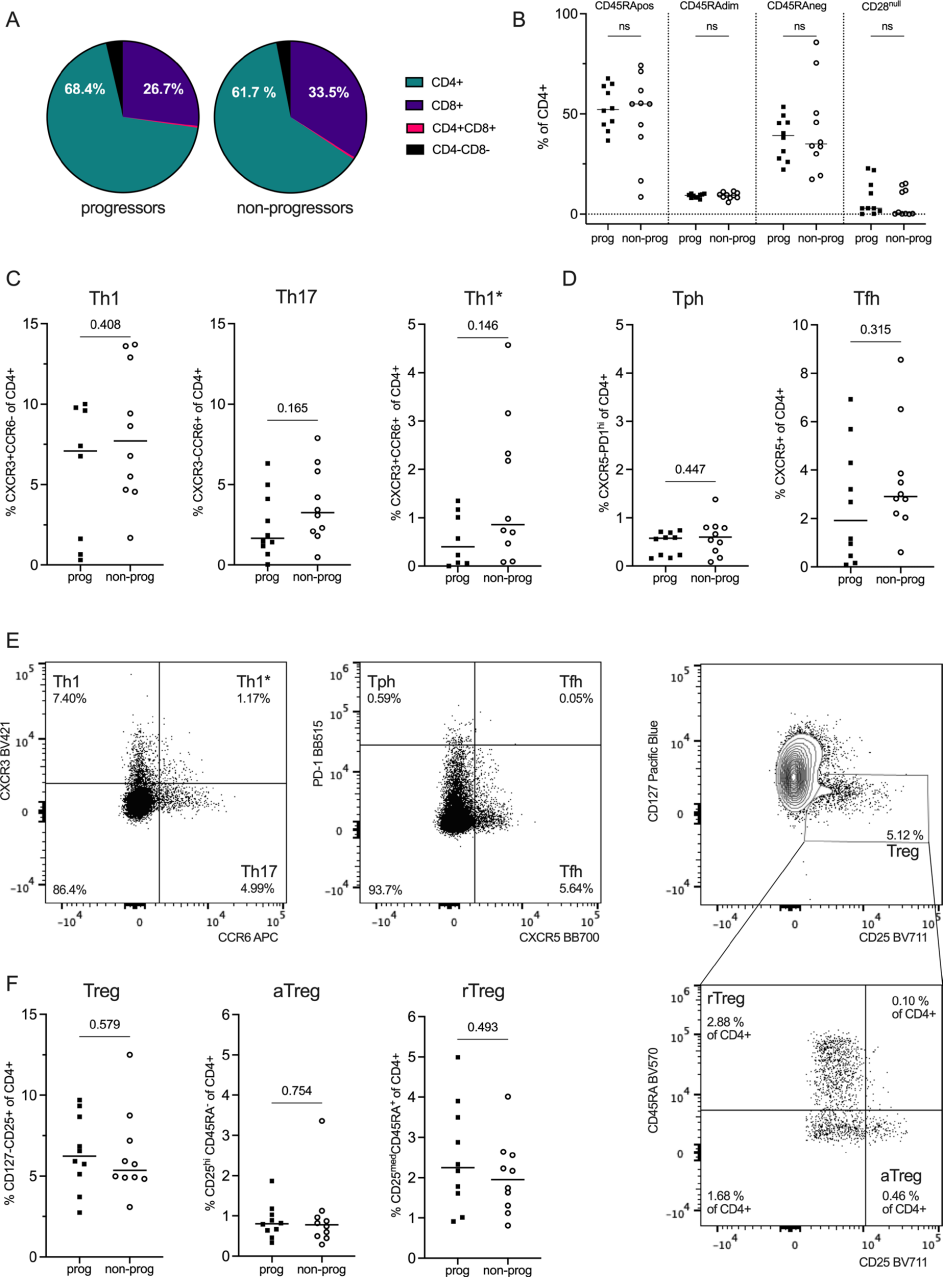
CD4+T cell subsets in progressors and non-progressors. (**A**) Median distribution of CD4+ and CD8+ T cells. (**B**) Frequency of naïve (CD45RApositive), intermediate (CD45RAdim) and memory subsets (CD45RAneg and CD28^null^) among CD4+T cells in the progressor and non-progressor groups. (**C, D**) Frequency of CD4+T-helper subsets including (**C**) Th1 (CXCR3+CCR6−), Th17 (CXCR3-CCR6+), Th1* (CXCR3+CCR6+) and (**D**) peripheral (Tph) (CXCR5-PD1hi) and T follicular helper (Tfh) (CXCR5+) cells in the progressor and non-progressor groups. Two individuals were excluded from the progressor Th1 and Th1* panels due to technical reasons. (**E**) Representative flow cytometry plots of T-helper subsets and subdivision of CD25+CD127− regulatory T cells (Treg) into activated (aTreg; CD45RA−CD25hi) and naive/resting (rTreg; CD45RA+CD25 med) T regulatory subsets. (**F**) Tregulatory subsets. Mann-Whitney U test.

T cell phenotyping was performed at all time points. Here, we present the baseline data. Median frequency of CD4+T cells was 68.4% in the progressor group (range 43.7–81.7) and 61.7% (range 17.0–77.3) in the non-progressor group (figure 2A, online supplemental figure 7A). Downstream analyses were performed within the CD4+compartment (ie, CD4+CD8−), where no significant differences were seen regarding frequencies of naïve (CD28+CD45RA+), memory (CD28+CD45RA− and CD28null) or CD28+CD45RAdim (intermediate CD45RA-expression) cells between the groups (figure 2B). Overall, EM cells were more frequent than CM cells in both groups (p<0.05, online supplemental figure 7B).

Th1 (CXCR3+CCR6−), Th1* (CXCR3+CCR6+) and Th17 (CXCR3-CCR6+) cells were seen in similar frequencies in the progressor and non-progressor group (figure 2C,E). Tph cells (CXCR5-PD-1hi) could be identified at very low frequencies in both groups (figure 2D,E). In addition, circulating Tfh cells (CXCR5+) were found in slightly higher frequencies than the Tph, although not significantly different between the groups (figure 2D). Tregs, assessed being CD127-CD25+, were seen in similar frequencies in both groups (figure 2E,F). Further analysis of the Tregs revealed that neither did the frequencies of activated aTregs (CD45RA-CD25hi) nor naïve/resting rTregs (CD45RA+CD25 med) differ between the groups (figure 2F). Notably, not all CD127-CD25+cells belonged to these subsets.^30 32^

### Antigen-specific T cells in the risk phase of RA

Antigen-specific CD4+T cells from all sample time points were assessed ex vivo using HLA-class II tetramers (figure 1B). Cells recognising citrullinated peptides presented by the HLA-DRB1*04:01 were rare but still found at baseline in all individuals. Of note, in HLA-DRB1*04:01 healthy donors (n=4) cit-specific CD4+T cells could also be detected (online supplemental figure 8A).

**Figure 3:**
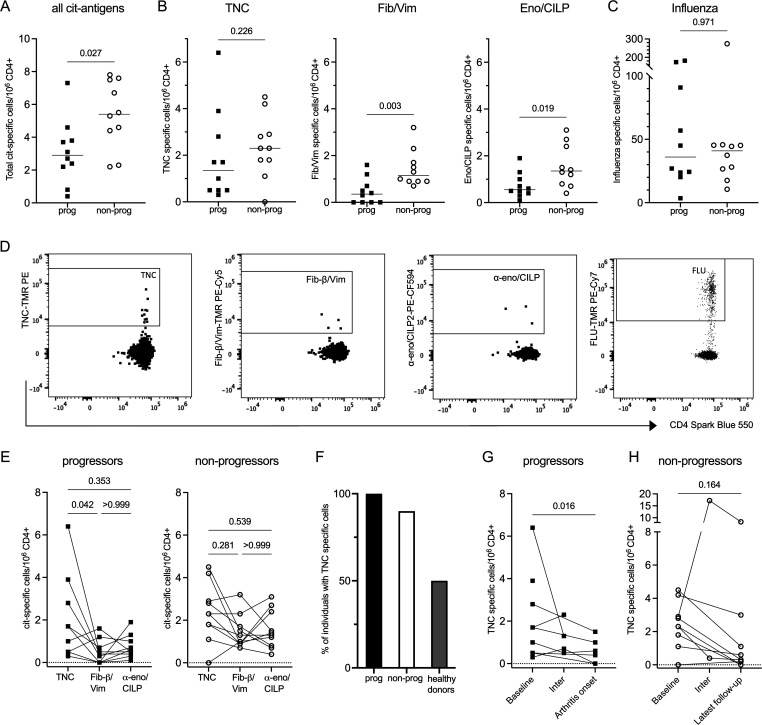
Antigen-specific CD4+T cells ex vivo. (**A**) Sum of citrulline-specific cells per million CD4+T cells. (**B**) Total number of cit-tenascin-C (TNC), cit-fibrinogen-β/vimentin and cit-α-enolase/CILP-specific cells per million CD4+T cells. (**C**) Total number of influenza-specific cells per million CD4+T cells (positive control). (**D**) Examples of tetramer staining for the different antigens (separate individuals). (**E**) Comparison of the frequencies of the different citrullinated antigen specificities among progressors (left panel) and non-progressors (right panel). (**F**) Percentage of individuals (progressors n=10, non-progressors n=10, healthy donors n=4) with cit-TNC-specific CD4+T cells. (**G, H**) Longitudinal assessment of cit-TNC-specific CD4+T cells from baseline to end-point (arthritis onset) or at latest follow-up time point (non-progressors). Squares=progressors, open circles=non-progressors in all graphs. (A–C): Mann-Whitney U test. (E) Friedman test. (G, H) Wilcoxon matched pairs test.

Intriguingly, such autoreactive T cells, although present in all individuals, were significantly less frequent in the progressor group than the non-progressor group (p<0.05) (figure 3A). This finding was consistent when looking separately at specific pools of antigens (ie, tetramer pools for citrullinated (cit) fibrinogen-β/vimentin and α-enolase/cartilage intermediate layer protein; CILP), between progressor and non-progressor individuals (p<0.01 and p<0.05, respectively) with a similar trend for cit-TNC-specific T cells (figure 3B). Whereas the anti-CCP2 levels were higher in the progressor group (table 1), the inverse relationship was seen in terms of frequency of circulating cit-specific CD4+T cells. In contrast, no difference was seen in the frequency of influenza-specific CD4+T cells between the groups (figure 3C). Flow plots showing positive tetramer staining for antigen-specific T cells (after Boolean gating) are shown in figure 3D.

We could observe individual differences in the patterns of preferential T cell cit-specificity, that is, the same individual did not have high (or low) frequencies towards all specificities. Within the progressor group the frequency of cit-TNC-specific T cells was significantly higher than the frequency of cit-fibrinogen-β/vimentin-specific T cells (p<0.05), but not compared with the frequency of cit-α-enolase/CILP-specific T cells (figure 3E, left panel). No significant differences were seen between the antigen-specificities in the non-progressor group (figure 3E, right panel) nor in the healthy donors (online supplemental figure 8B). Notably, only 50% (n=2) of the healthy donors had detectable cit-TNC-specific T cells in contrast to 100% of progressors and 90% of non-progressor individuals (figure 3F).

The longitudinal design of the Risk-RA cohort allowed us to also study whether the frequencies of antigen-specific T cells changed during follow-up. Interestingly, there was a significant decrease of cit-TNC-specific CD4+T cells in the progressor group on arthritis onset (p<0.05) (figure 3G). For three individuals (out of seven), it was not possible to detect cit-TNC-specific CD4+T cells at arthritis onset (figure 3G). A similar trend, although not significant, was observed within the non-progressor group from baseline to the latest follow-up time point (figure 3H). No decrease in frequency was seen over time for influenza, cit-fibrinogen-β/vimentin or cit-α-enolase/CILP-specific cells in any of the groups (online supplemental figure 9A–F).

### Phenotype of citrulline-specific CD4+ T cells at baseline

**Figure 4:**
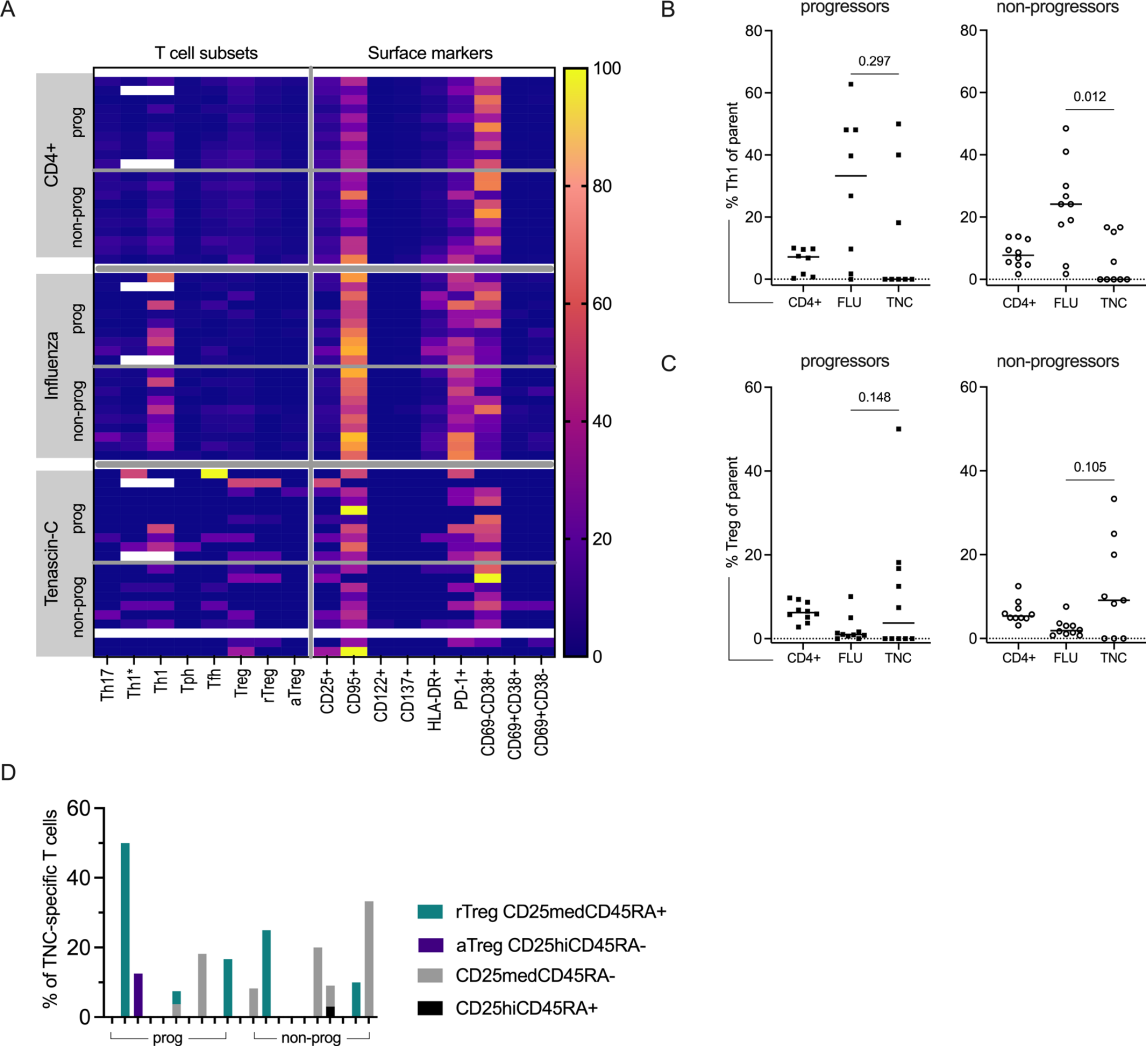
T cell subsets and surface phenotype in CD4+T cells and antigen-specific T cells. (**A**) Heatmap showing frequencies of T cell subsets and surface markers in the global CD4+ (upper panel), influenza (middle panel) and cit-tenascin-C (cit-TNC) (lower panel) compartment in progressor and non-progressor individuals respectively. One non-progressor individual lacked cit-TNC cells (white line) and n=2 progressor individuals were excluded from Th1, Th1* analysis due to technical reasons (also excluded in B). (**B, C**) Frequency of Th1 (CXCR3+CCR6−) (**B**) and Treg (CD25+CD127−) (**C**) subsets. (**D**) Subdivision of CD25+CD127 dim Tregs in activated (aTreg), naive/resting (rTreg) and non-Treg subsets. Each bar represents one individual. Wilcoxon matched-pairs signed rank test.

We next assessed whether the phenotype of the citrulline-specific CD4+T cells differed between the progressor and non-progressor individuals also in comparison to influenza-specific cells and the global CD4+T cell compartment (figure 4A). Given the rarity of the citrulline-specific cells, especially within the progressor group, we focused our downstream analysis on the most frequent reactivity — cit-TNC-specific T cells (median cell count progressors: 4.5, range 1–27, non-progressors: 6, range 0–33) (online supplemental figure 9G).

In the influenza compartment, we observed an increased signal for Th1 (CXCR3+CCR6−) cells (figure 4A). In particular, this was seen in comparison to the cit-TNC-specific CD4+T cells in the non-progressor group (p<0.05) with a similar trend in the progressor group (figure 4B). In the cit-TNC compartment, we also detected a tendency towards an increased Treg (CD127-CD25+) phenotype at baseline as compared with influenza (figure 4C). Whereas aTregs were rarely found (only in one individual), there were rTregs among the cit-TNC-specific cells. Notably, within the non-progressor group four of six individuals had neither cit-TNC-specific aTregs nor rTregs on such subphenotyping (figure 4D). To summarise, no distinct T cell subset dominated among the cit-TNC-specific cells (figure 4A and online supplemental figure 10A). We then wanted to investigate if the higher frequency of cit-fibrinogen-β/vimentin and cit-α-enolase/CILP-specific cells in the non-progressor group could be explained by a Treg phenotype of these cells and thus potentially providing a regulatory advantage in these individuals. Although all non-progressor individuals had cells recognising these specificities, the majority of individuals did not have any Tregs among these cit-specific cells (online supplemental figure 10B).

**Figure 5:**
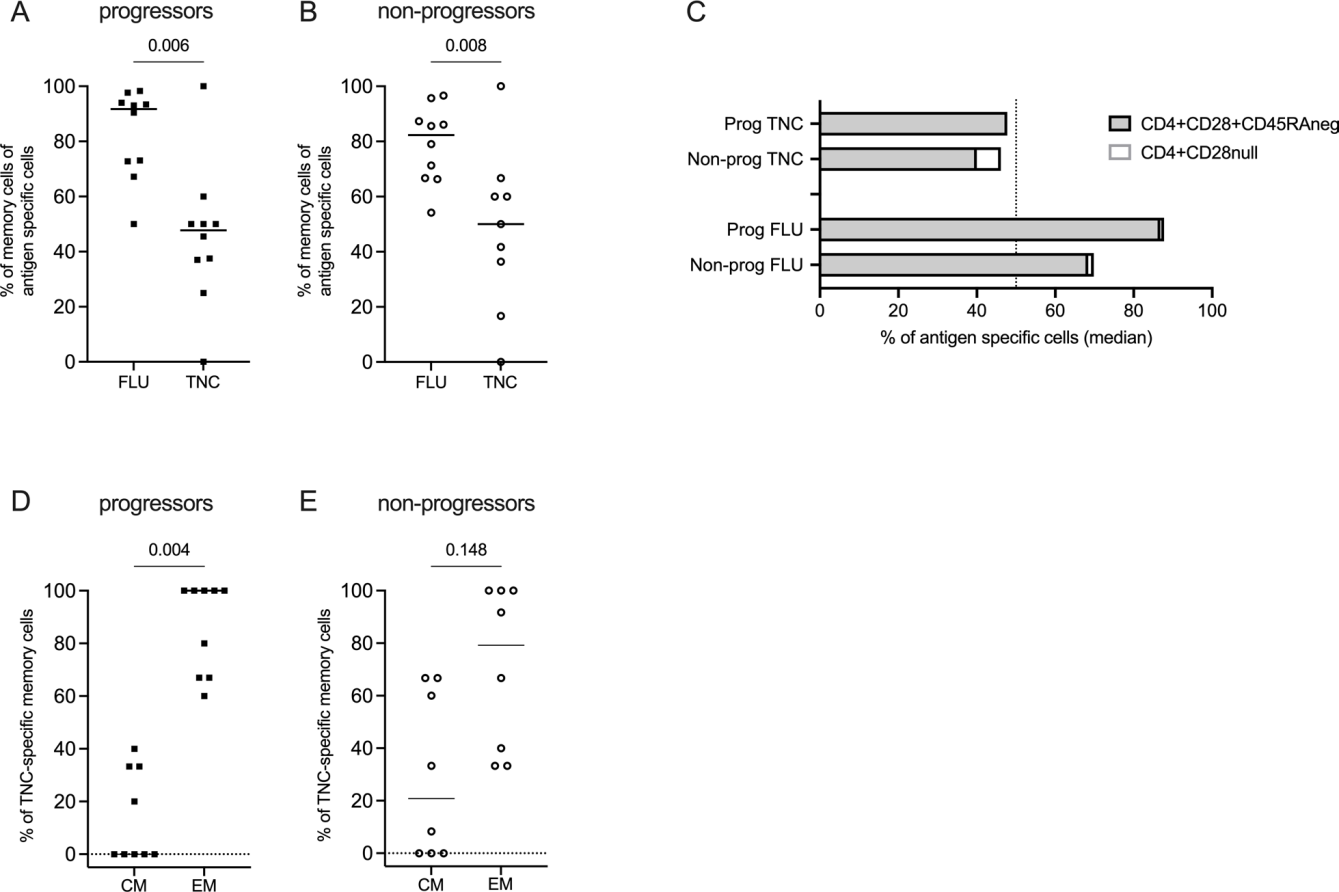
Distribution of antigen-specific memory cells and their subsets. (**A, B**) Frequency of antigen-specific (cit-tenascin-C or influenza) with memory phenotype, that is, being CD28+CD45RA- or CD28^null^ in progressor and non-progressor individuals. (**C**) Median frequency of CD28+CD45RA- and CD28^null^ antigen-specific cells, respectively. (**D, E**) Frequency of central memory (CM) and effector memory (EM; including EM cells re-expressing RA) of cit-tenascin-C-specific memory cells. Wilcoxon matched-pairs signed rank test. Squares=progressors, open circles=non-progressors in all graphs. RA, rheumatoid arthritis.

When further investigating the activation phenotype, expression of both CD95 and PD-1 was pronounced in the influenza compartment, whereas CD69-CD38+phenotype was seen in the global CD4+compartment and the cit-TNC compartment. No striking differences were seen between the progressors and non-progressors at this level (figure 4A). To further dissect which cells were expressing these receptors, we subdivided them based on their memory (or naïve) phenotype, followed by assessment of activation marker expression (online supplemental figure 11). The influenza-specific CD4+T cells were primarily of memory phenotype (CD28+CD45RA− or CD28null), with a significantly higher frequency than the cit-TNC-specific cells (p<0.05 in both groups; figure 5A–C). The majority of individuals with cit-TNC-specific cells (9/10 in progressors and 8/9 in non-progressors, respectively) had cells with a memory phenotype, indicating a prior encounter with the antigen. Notably, whereas we observed an EM over CM dominance within the progressor group (p<0.01), this was not as evident in the non-progressor group (figure 5D,E). Influenza-specific memory T cells were primarily of effector phenotype in both groups (online supplemental figure 12A, B).

**Figure 6:**
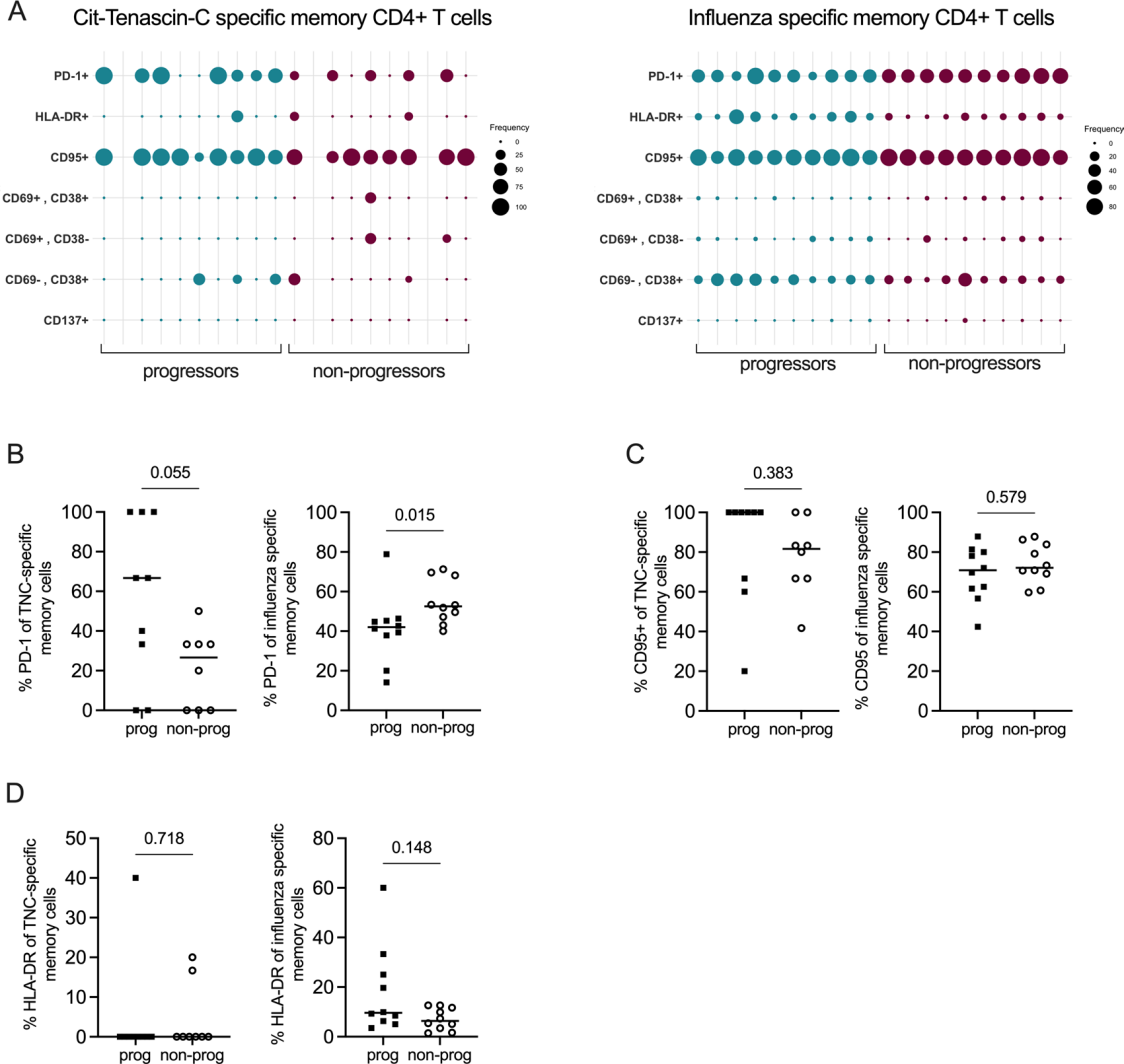
Phenotype of antigen-specific memory T cells. (**A**) Bubble plot showing the frequencies of surface marker expression on cit-tenascin-C (left panel) and influenza-specific (right panel) memory (CD28+CD45RAneg or CD28^null^) T cells. (**B–D**) Frequency of PD-1 (**B**) CD95 (**C**) and HLA-DR (**D**) expression on cit-tenascin-C (left panels) and influenza-specific (right panels) memory T cells in progressor and non-progressor individuals. Mann-Whitney U test.

Among the antigen-specific memory T cells, PD-1 and CD95 expression was evident both in the cit-TNC-specific and influenza-specific subsets (figure 6A). There was a clear tendency of a higher frequency of PD-1 positivity among cit-TNC-specific T cells in the progressor group, compared with the non-progressor group (p=0.055; figure 6B), whereas the opposite was seen in the influenza compartment (p<0.05). Although the majority of cit-TNC-specific memory cells in the progressor group were CD95+, this was not significantly different compared with the non-progressor group, nor among influenza-specific cells (figure 6C). Contrary to influenza-specific cells, the expression of HLA-DR and CD38 in memory cit-TNC CD4+T cells was confined to only a few individuals, while being commonly expressed in the former cell population (figure 6A,D). CD137 was expressed on a small subset of CD4+CD45RA− cells in the global compartment (data not shown), but not at all on cit-TNC-specific memory cells (figure 6A).

## Discussion

In this study, we have explored the presence and phenotype of cit-specific autoreactive CD4+T cells in the development of RA during the period leading up to arthritis onset (the pre-RA phase) and compared this with group of at-risk individuals who have not (yet) progressed to disease.

Although rare, we could detect autoreactive cit-specific CD4+T cells in all individuals. Surprisingly, the frequency of such cells was lower at baseline in the progressor group raising the question whether this reflects an ongoing homing of autoreactive cells to the joint or joint draining lymphoid structures. Although currently unknown, the initial encounter of citrulline-specific autoreactive T cells with their cognate antigen is thought to take place at, or derive from mucosal sites, as suggested by the identification of cit-reactive B cells in the gums and lungs of at-risk individuals.^1 33 34^ We hypothesise that reactivation of cit-specific T cells in progressor individuals is likely to take place in closer proximity to the joint as the individuals experiences musculoskeletal symptoms. The decrease of the most frequently observed cit-reactivity (cit-TNC-specific T cells) from baseline to arthritis onset lends support to this hypothesis, particularly when considering our prior observations that cit-TNC-specific cells are prevalent in the synovial fluid of patients with established disease.^17 28^ While analysing synovial fluid from larger joints is feasible in later stages of RA, identification of cit-specific T cells in a non-yet inflamed joint would be challenging. Previous studies have though suggested that lowering of, for example, naïve T cells and Tregs in periphery and presence of T cells in synovia is predicting disease.^6 23 35^

A trend with a decreased frequency of cit-TNC-specific cells was also seen, during the longer clinical observational time between sampling within the non-progressor group. We hypothesise that this decrease is due to other mechanisms, possibly reflected by the higher relative proportion of cit-TNC-specific CM T cells within this group which could suggest that these cells home to non-articular mucosal lymphoid structures. The narrower antibody reactivity towards citrullinated peptides in this subgroup also indicates that these individuals have less evolved autoimmune responses.

In contrast, the frequencies of cit-α-enolase/CILP and cit-fibrinogen-β/vimentin-specific T cells were higher in the non-progressor group already at baseline and this did not change over time, neither in the non-progressor nor progressor group, suggesting different kinetics of these specificities. Although the absence of a cell subset is difficult to evaluate, the low levels of cit-fibrinogen-β/vimentin and cit-α-enolase/CILP specific T cells among the progressor group could possibly represent a steady-state level that results from an earlier homing event not captured in our study. Notably, CD4+T cells recognising the above-mentioned antigens and cit-vimentin-specific T cells in particular, can be found in the lymph nodes of at-risk individuals and early RA patients.^16^ The parallel finding of a broad antibody reactivity towards citrullinated peptides in the progressor group further underscores a previous T-B cell interaction in this group.

The occurrence of cit-specific CD4+T cells in the at-risk phase, including citrullinated CILP, enolase, vimentin and fibrinogen, has recently been reported also by James *et al*. Although there are methodological differences between our studies, the frequencies of these cit-specificities are within a similar range as our results—including the observation that cit-vimentin/fibrinogen are found at lower frequencies.^21^ Whereas James *et al* had the advantage of studying many individuals, our study provides unique and novel data with our longitudinal sampling and clinical data on disease progression.

Screening for virus-reactive T cells makes an interesting comparison for autoreactivity since the nature of the antigen is strikingly different both during the infection phase (with parallel pathogen-associated molecular patterns/toll-like receptor activation) as well as its subsequent disappearance. As expected, the influenza-specific T cells were primarily of memory phenotype,^13 16 18 36^ whereas only 50% of the cit-TNC-specific cells displayed a memory phenotype (similar to the global CD4+compartment). Additionally, influenza-specific cells displayed a clear bias towards CXCR3 positivity, a feature previously reported also for citrulline-specific T cells in established RA,^13^ which we did not see in this at-risk population. Neither did we see any dominance of Th17, Th1*, Tph or Tfh among the cit-TNC-specific cells. Instead, we could show that a subset of the cit-TNC-specific T cells displays a Treg phenotype (primarily CD45RA+rTregs). However, the increased frequency of cit-specific cells in the non-progressor group was not explained by a dominant Treg phenotype in any of the specificities. It could thus be speculated that (1) the Treg cit-TNC-specific cells represent a subset with tolerogenic features; (2) these cells have an impairment in Treg function and therefore accumulate as such or (3) their presence reflects an initiated inflammatory event with ongoing peripheral Treg differentiation. The trend of higher frequency of PD-1+memory cit-TNC-specific cells among the progressor individuals, also in combination with CD95 expression, might represent a repeated antigen exposure, but in contrast to established disease,^17^ CD38+memory cit-TNC-specific cells were not a frequent finding in our study. The PD-1 expression is interesting also from a targeting perspective, as treatment with peresolimab, a monoclonal antibody that binds and activates PD-1, has recently been tested in a phase 2 trial to treat RA.^37^

Whereas more pronounced phenotypic differences between the antigen-specific CD4+T cells could have been anticipated, it is notable that we do detect trends even when studying small number of cells. As mentioned in the previous section the phenotype of the cit-TNC-specific cells did differ from what has previously been shown for cit-specific cells in established disease, thus suggesting differences of T cell phenotype from one specificity to another and during the time course of disease.

Comparison of the global CD4+T cell compartment between the groups revealed a similar phenotype in regard to T helper, Treg as well as naïve/memory subsets. The limited number of individuals available in our groups is a plausible explanation for why this differs from prior studies,^22 23^ another being that we have studied a subset of individuals (both progressors and non-yet-progressors) that are immunologically homogeneous in terms of carrying the RA-associated HLA-DRB1*04:01 allele and the presence of autoantibodies. Notably, due to these factors and musculoskeletal complaints, the non-progressors individuals might still have a risk of future development of RA. On the contrary, there were differences at baseline sampling among the groups in terms of RF-positivity, titres of ACPA as well as having a double shared epitope allele, reflecting our prior characterisation of the Karolinska risk-RA cohort showing these factors as major risks for disease progression.^8^

Usage of HLA-class II tetramers poses a limitation to studies like this, not only because all individuals will have a strong genetic risk factor for RA, but also because it limits the available number of individuals to be included. However, HLA-class II tetramers have made it possible to identify very rare antigen-specific CD4+T cells. Although one needs to be cautious with interpretations due to the low cell numbers assessed, we hope to inspire fellow researchers to pursue further studies to extend this knowledge.

To conclude, we show that cit-specific CD4+T cells are present in individuals at risk of developing RA and that a fraction of such citrulline specific cells display phenotypes that suggest a previous engagement with their cognate antigen. Intriguingly, the frequencies of cit-reactive CD4+T cells were lower in individuals who later progressed to arthritis, which might represent a process of homing of these cells from the circulation to the joint and/or joint-associated lymphoid tissue. The occurrence of cit-TNC specific CD4+ T cells has not been previously investigated in the at-risk phase, where our results further strengthen cit-TNC as an interesting T cell antigen in RA. Lastly, the identification of still naïve cit-specific CD4+T cells in at-risk individuals suggests the existence of a window of opportunity for therapeutic intervention, with the aim of restoring immune tolerance.

## supplementary material

10.1136/rmdopen-2024-004510 online supplemental file 1

10.1136/rmdopen-2024-004510 online supplemental file 2

## Data Availability

Data are available on reasonable request.
