# Supplementary material for: Arthritis progressors have a decreased frequency of circulating autoreactive T cells during the at-risk phase of rheumatoid arthritis
Source: RMD Open. 2024 Nov 18;10(4):e004510. doi: 10.1136/rmdopen-2024-004510 (PMC11574433; doi:10.1136/rmdopen-2024-004510)
Supplement: online supplemental file 1 [file rmdopen-10-4-s001.pdf]

Supplementary figures

Arthritis progressors have a decreased frequency of circulating autoreactive T cells during the at-risk phase of rheumatoid arthritis

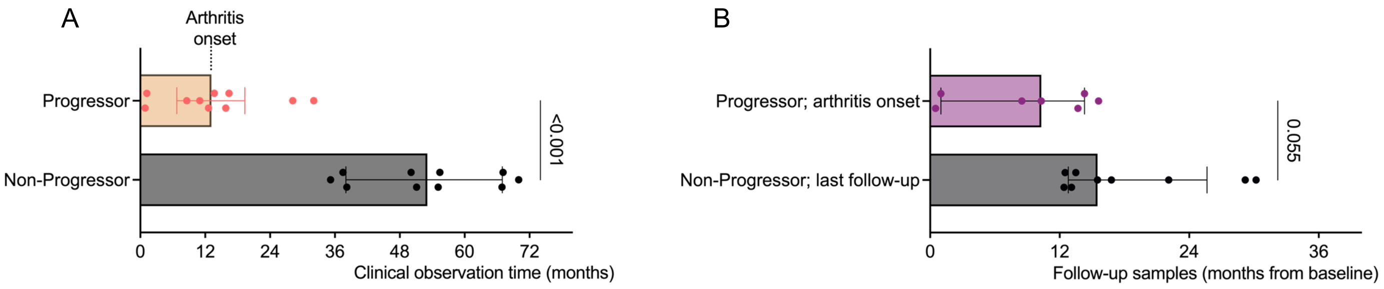

Supplementary fig. 1. Clinical follow-up and time of sampling

A. The individuals in the cohort are followed to arthritis onset (end-point) or for at least 36 months. Bars indicating median time to end-point (e.g. time to progression) or total clinical follow-up time within the study period (non-progressors).

B. Blood samples are collected at baseline and follow-up visits. Bars indicating median time to sampling at arthritis onset (progressors) and the last available follow-up sample from the non-progressors. Whiskers = interquartile range in A and B. Mann-Whitney test in A and B.

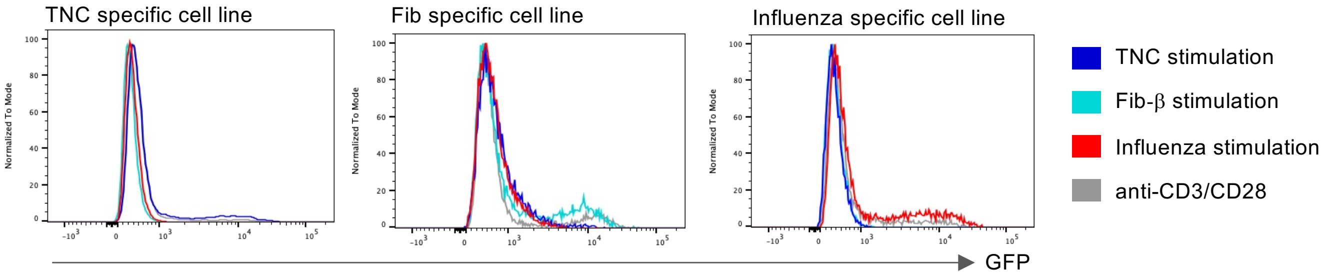

Supplementary fig. 2. **Testing specificity for peptide-HLA monomer.** Specificity of peptide-HLA monomers were tested using antigen specific T cell lines recognizing citrullinated tenascin-C ($TNC_{1012\text{-}1026}$), fibrinogen β($fib_{69\text{-}81}$), or influenza ($MP97_{97\text{-}116}$) peptides also expressing a NFAT-GFP reporter. Anti-CD3/CD28 stimulation was used as positive control. TCR signalling resulting in GFP expression was assessed by flow cytometry.

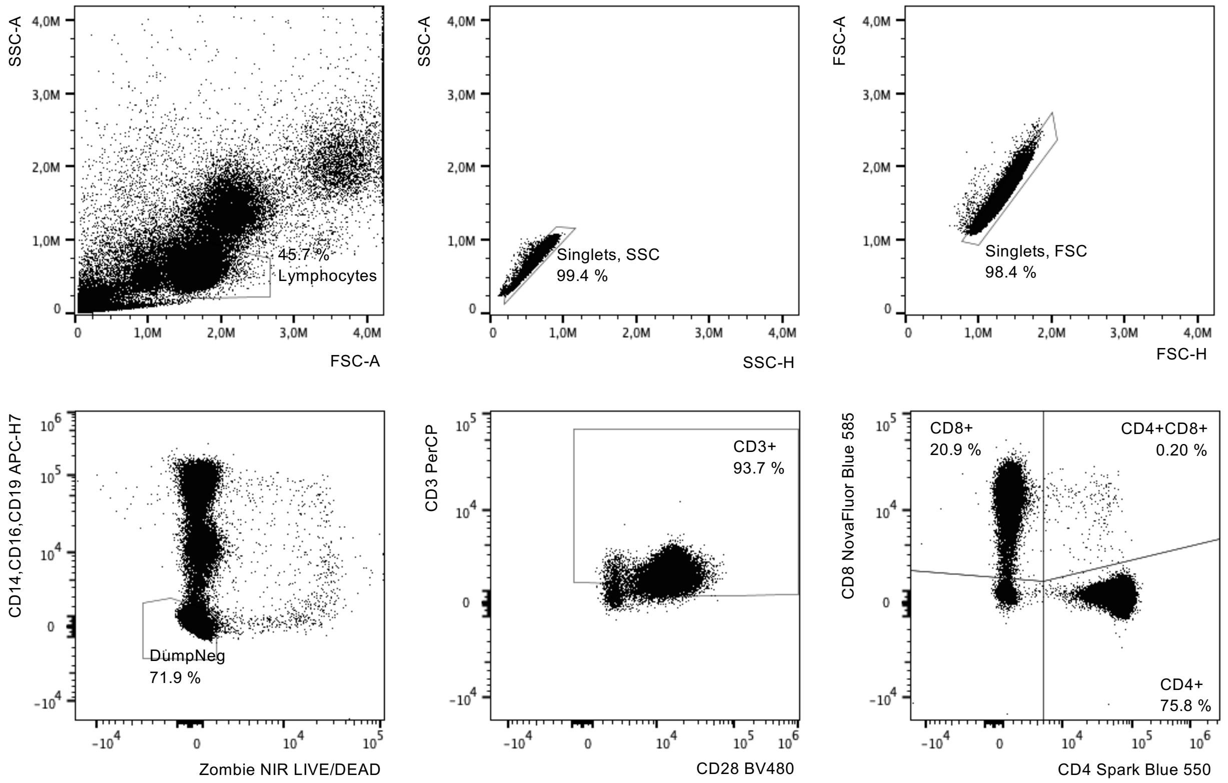

Supplementary fig. 3. Gating strategy

Representative presentation of the gating strategy. All downstream analyses are performed within the CD4+CD8- compartment.

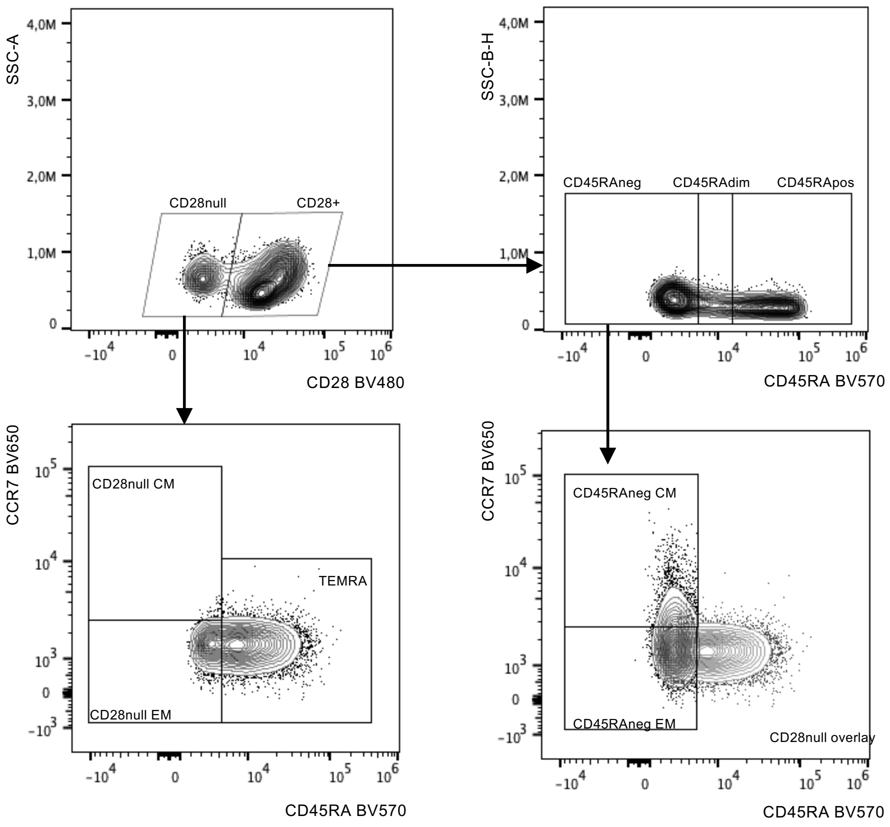

Supplementary fig. 4. Gating strategy for memory cells

Representative presentation of gating strategy. Memory status was defined by being either CD28null, or CD28+CD45RAneg. CD45RAdim cells were not included in neither memory nor naive subsets to have as stringent gating as possible. The gate dividing central- and memory CD28null cells was used to aid gating of these subsets in the CD45RAneg compartment (here overlaid as an example). Additionaly CD45RAvsCCR7 staining in the CD8+ compartment was also used to determine the gate as CCR7 separated better in that setting (not shown).

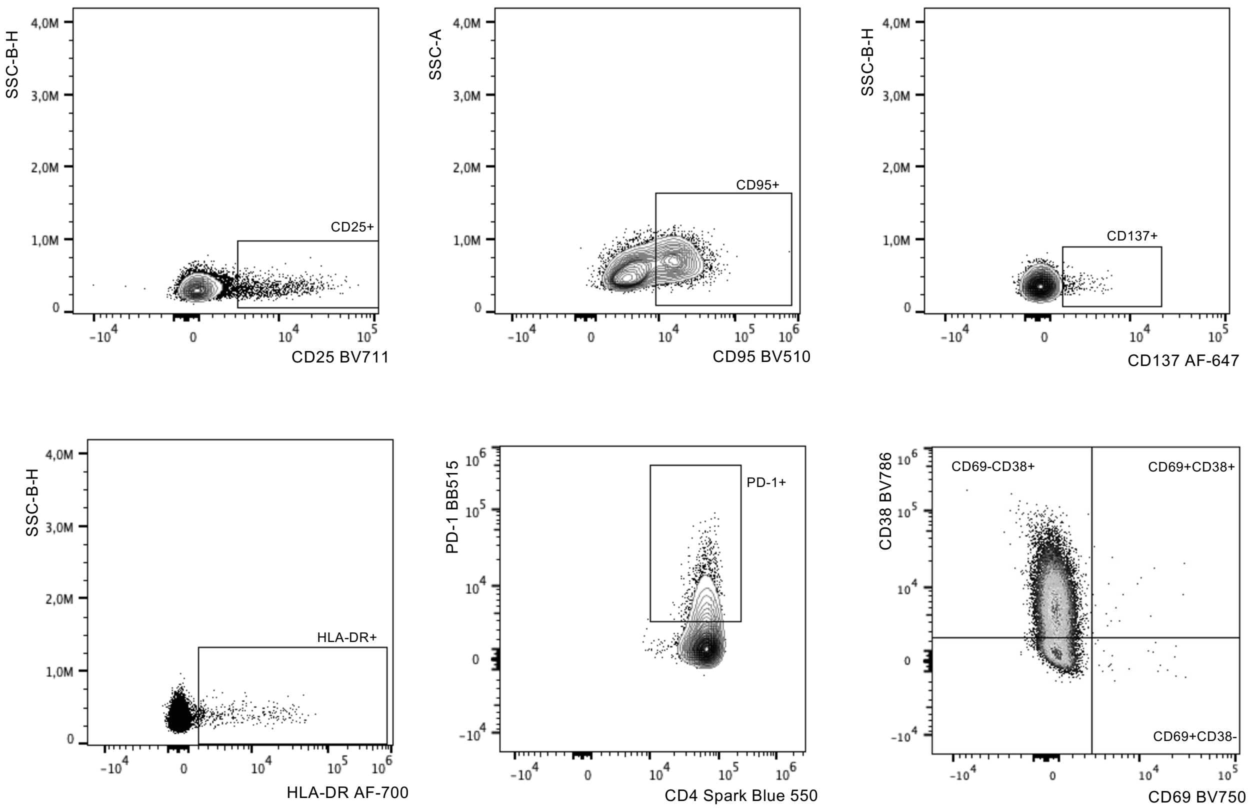

Supplementary fig. 5. Gating strategy for activation markers in CD4+ cells

Representative plots for CD25, CD95, CD137, HLA-DR, PD-1 and CD38vsCD69 gating.

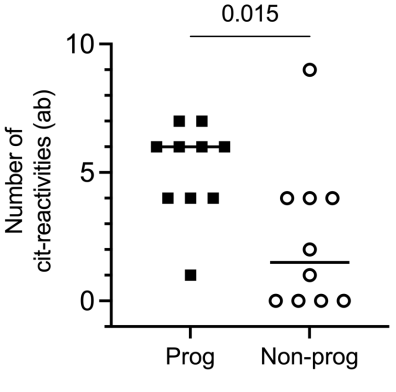

Supplementary fig. 6. Serology at baseline cell sampling

Number of fine-specific antibody reactivities towards citrullinated antigens per individual. Having one reactivity correspond to a value above cut-off for that specific antigen. Squares = progressors, open circles = non-progressors. N=9 citrullinated antigens included as indicated below. Mann-Whitney test.

Tenascin-C:
- $Cit_{(2033)}$-$TNC_{2025\text{-}2040}$
- $Cit_{(2187, 2192, 2197, 2198)}$-$TNC_{2177\text{-}2200}$

Fibrinogen-β:
- $Cit_{(44)}$-$Fib\beta_{36\text{-}52}$
- $Cit_{(60, 72, 74)}$-$Fib\beta_{60\text{-}74}$

Vimentin:
- $Cit_{(64,69,71)}$-$Vim_{60\text{-}75}$

Filaggrin:
- $Cit_{(9)}$-$Fil_{7\text{-}27}$

Histone3:
- $Cit_{(2, 8,17, 26)}$-$His3_{1\text{-}30}$
- $Cit_{(26, 40, 42)}$-$His3_{21\text{-}44}$

Fibrinogen-α:
- $Cit_{(38,42)}$-$Fib\alpha_{36\text{-}50}$

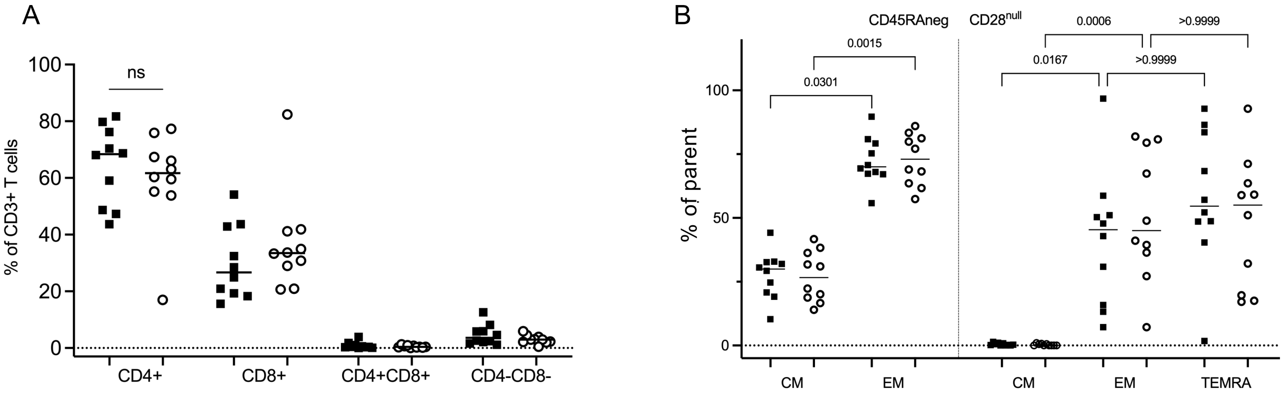

Supplementary fig. 7. T cell and memory subset distribution

A. Distribution of CD4+, CD8+, CD4+CD8+ and CD4-CD8- cells in progressors (squares), non-progressors (open circles). Mann-Whitney test. **B.** Distribution of the CD4+ memory subsets in progressors (squares), non-progressors RA (open circles). CM= central memory, EM = effector memory, TEMRA = effector memory re-expressing RA. Friedman test, Dunn's multiple correction.

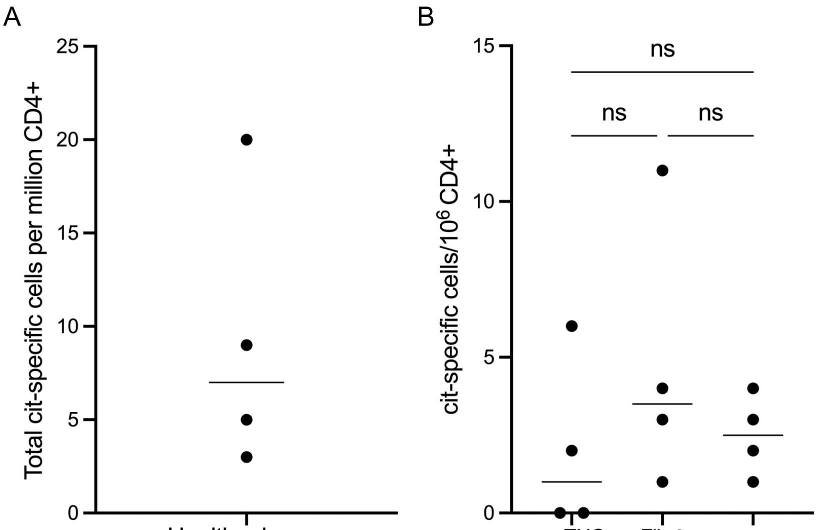

Supplementary fig. 8. Cit-specific CD4+ T cells in healthy donors

A. Total cit-specific cells per million CD4+ T cells in healthy donors. **B.** cit-specific cells per specificity (TNC = tenascin-C, Fibrinogen/vimentin, enolase/CILP) in healthy donors. Friedmans test.

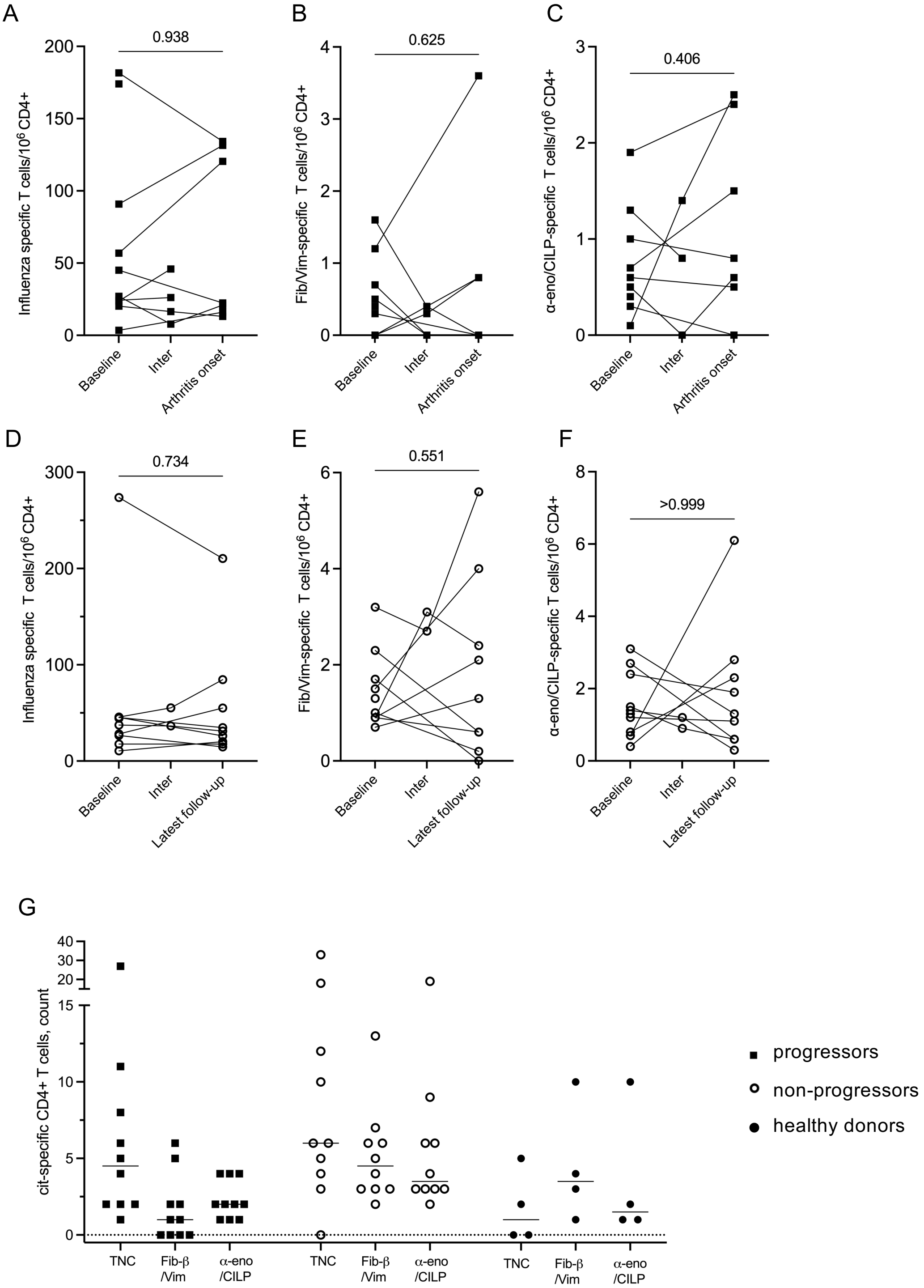

Supplementary fig. 9.

A-F. Frequencies of influenza specific, cit-fibrinogen-β/vimentin and cit-α-enolase/CILP reactive cells during follow-up in progressor (A, B, C) and non-progressor individuals (D, E, F). **G.** Absolute count of cit-specific T cells at baseline. Squares = progressors, open circles = non-progressors and filled circles = healthy donors.

A

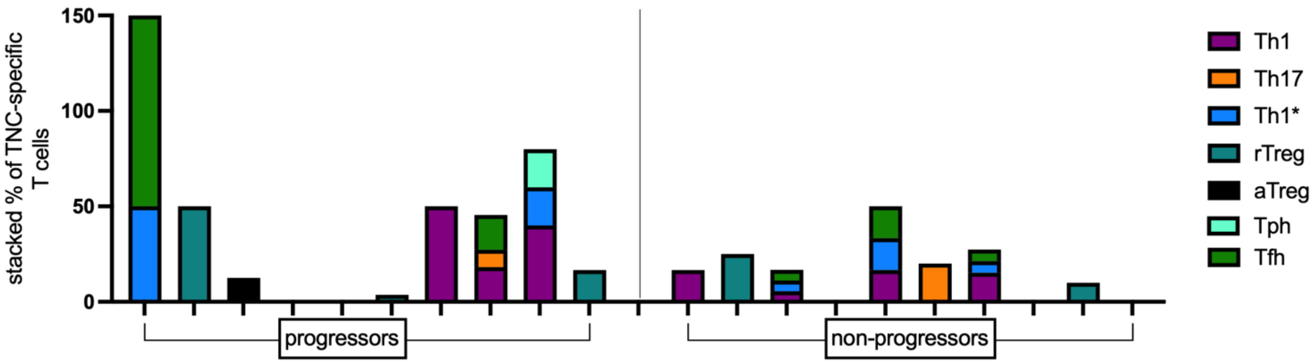

B

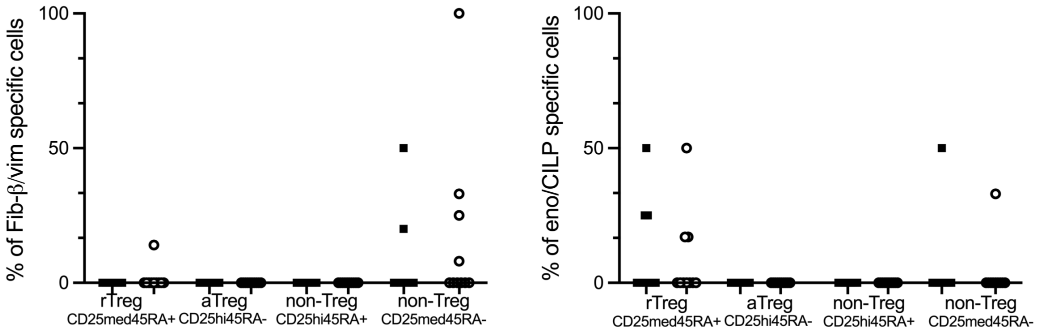

Supplementary fig. 10.

A. T cell subsets in cit-TNC specific cells (stacked). Each bar represents an individual. The Treg, Tph/Tfh and Th1/Th17 phenotypes have been gated separately on CD4+ cells, hence the same cells can be represented more than once in this graph (e.g. 150 % in the first column).

B. Treg sub phenotype in cit-fibrinogen-β/vimentin and cit-α-enolase/CILP reactive cells at baseline. Squares = progressors, open circles = non-progressors in all graphs. Wilcoxon matched pairs test.

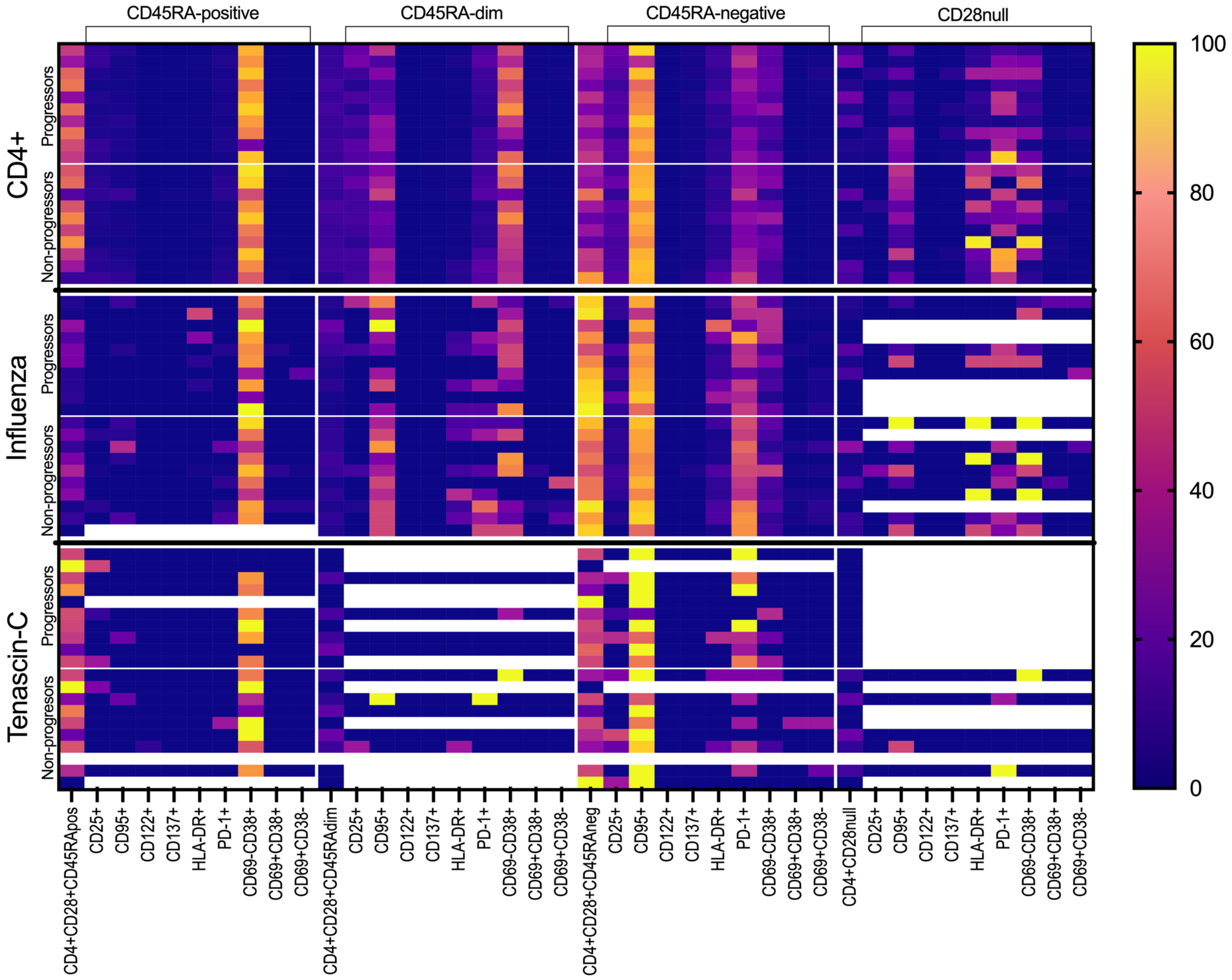

Supplementary fig. 11.

Phenotypic markers in naive, CD45RAdim and memory subsets in cit-TNC, influensa specific and CD4+ T cells in progressors and non-progressors.

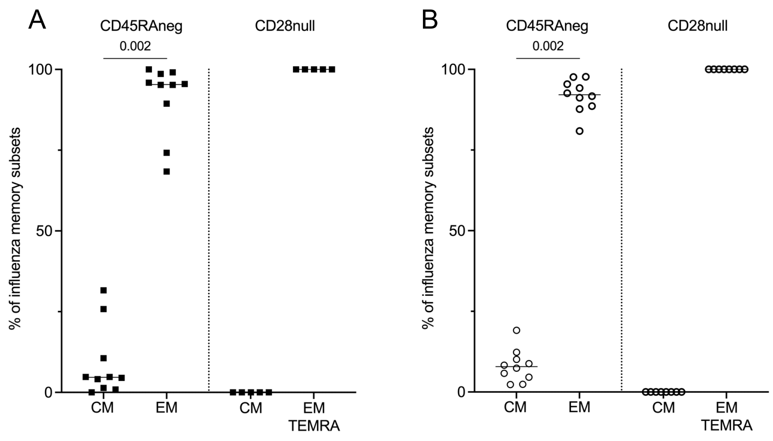

Supplementary fig. 12.

Distribution of central memory (CM), effector memory (EM) and T cells re-expressing RA (TEMRA) subsets in influenza specific memory cells; CD45RAneg and CD28null respectively. Progressor group in A (squares) and non-progressor group in B (open circles). Wilcoxon matched pairs test.
