# Supplementary material for: Arthritis progressors have a decreased frequency of circulating autoreactive T cells during the at-risk phase of rheumatoid arthritis
Source: RMD Open. 2024 Nov 18;10(4):e004510. doi: 10.1136/rmdopen-2024-004510 (PMC11574433; doi:10.1136/rmdopen-2024-004510)
Supplement: online supplemental file 2 [file rmdopen-10-4-s002.pdf]

Arthritis progressors have a decreased frequency of circulating autoreactive T cells during the at-risk phase of rheumatoid arthritis

Supplementary material and methods

Supplementary table 1. Peptides used in assembly of HLA-class II tetramers

Fluoro-chrome	Peptide$_{position}$	Protein	Sequence (citrulline = X)	Provider	Purity
PE	TNC17$_{871-885}$	tenascin-C	871-VSLIS**XX**GDMSSNPA	Merck-Sigma	97.90%
PE	TNC22$_{1012-1026}$	tenascin-C	1012-FDXY**X**LNYSLPTGQW	Merck-Sigma	97.70%
PE	TNC45$_{1633-1647}$	tenascin-C	1633-PDGF**X**LSWTADEGVF	Merck-Sigma	96.50%
PE	TNC56$_{2067-2081}$	tenascin-C	2067-QGQYEL**X**VDL**X**DHGE	Merck-Sigma	95.00%
PE-CF594	α-eno$_{11-25}$	α-enolase	11-IFDS**X**GNPTVEVDLF	BioS&T	>89.65%
PE-CF594	α-eno$_{26-40}$	α-enolase	26-TSKGLF**X**AAVPSGAS	GenScript	98.10%
PE-CF594	α-eno$_{326-340}$	α-enolase	326-K**X**IAKAVNEKSCNCL	GenScript	98.90%
PE-CF594	CILP$_{297-311}$	cartilage intermediate layer protein (CILP)	297-ATIKAEFV**X**AETPYM	GenScript	98.60%
PE-Cy5	fib$_{70-81}$	fibrinogen-β	70-GY**X**A**X**PAKAAAT	GenScript	98%
PE-Cy5	fib$_{69-81}$	fibrinogen-β	69-GGYRA**X**PAKAAAT	GenScript	97.10%
PE-Cy5	vim$_{59-78}$	vimentin	59-GVYAT**X**SSAV**X**L**X**SSVPGVR	GenScript	95.20%
PE-Cy5	vim$_{418-431}$	vimentin	418-FSSLNL**X**ETNLDSL	GenScript	99.70%
PE-Cy7	MP97$_{97-116}$	influenza matrix protein	97-YRKLKREITFHGAKE	GenScript	99.90%

Supplementary table 2. Antibodies used for flowcytometry

Surface marker	Fluorochrome	Clone	Company
**CD3**	PerCP	SP34-2	BD
**CD4**	Spark-Blue 550	SK3	Biolegend
**CD8**	NovaFluor Blue 585	OKT8	ThermoFisher
**CD25**	BV711	BC96	Biolegend
**CD28**	BV480	CD28.2	BD
**CD38**	BV785	HIT2	BD
**CD69**	BV750	FN50	BD
**CD95**	BV510	DX2	BD
**CD122**	PerCP-eFluor710	TU27	ThermoFisher
**CD127**	Pacific Blue	A019D5	Biolegend
**CD137**	Alexa-Fluor647	4B4-1	Biolegend
**CCR6**	APC	11A9	BD
**CCR7**	BV650	3D12	BD
**CXCR3**	BV421	G025H7	Biolegend
**CXCR5**	BB700	RF8B2	BD
**PD-1**	BB515	EH12.1	BD
**HLA-DR**	Alexa-Fluor700	G46-6	BD
**CD45RA**	BV570	HI100	Biolegend
**CD14**	APC-H7	MφP9	BD
**CD16**	APC-H7	3G8	BD
**CD19**	APC-H7	HIB19	BD
**ZOMBIE Near IR LIVE/DEAD Viability dye**			Biolegend
